# Risk of Infective Endocarditis Associated with Transcatheter Aortic Valve Implantation versus Surgical Aortic Valve Replacement: A Propensity Score-Based Analysis

**DOI:** 10.3390/jcm12020586

**Published:** 2023-01-11

**Authors:** Jorge Calderón-Parra, Juan E. de Villarreal-Soto, Juan Francisco Oteo-Domínguez, María Mateos-Seirul, Elsa Ríos-Rosado, Laura Dorado, Beatriz Vera-Puente, Carlos Arellano-Serrano, Antonio Ramos-Martínez, Alberto Forteza-Gil

**Affiliations:** 1 Infectious Diseases Unit, Service of Internal Medicine, Hospital Universitario Puerta de Hierro, 28222 Majadahonda, Spain; 2 Research Institute Puerta de Hierro-Segovia de Aranda, 28009 Madrid, Spain; 3 Cardiovascular Surgery Department, Hospital Universitario Puerta de Hierro, 28222 Majadahonda, Spain; 4 Interventional Cardiology Unit, Cardiology Department, Hospital Universitario Puerta de Hierro, 28222 Majadahonda, Spain

**Keywords:** infective endocarditis, TAVI, risk factors

## Abstract

Background: Infective endocarditis (IE) is a feared complication after surgical aortic valve replacement (SAVR)/transcatheter aortic valve implantation (TAVI). It is not certain which procedure carries a higher risk. Our aim was to assess the risk of IE after SAVR/TAVI. Methods: We conducted an observational study of a prospective cohort, including patients with TAVI/SAVR, from March 2015 to December 2020. IE was defined according to the modified Duke’s criteria. IE occurring during the first 12 months of the procedure was considered early IE, and an episode occurring after 12 months was considered late IE. The propensity score was designed to include variables previously associated with TAVI/SAVR and IE. An inverse probability of treatment weight was generated. Results: In total, 355 SAVR and 278 TAVI were included. Median follow-up, 38 vs. 41 months, *p* = 0.550. IE occurred in 5 SAVR (1.41%, 95% CI 0.2–2.6) vs. 13 TAVI (4.65%, 95% CI 2.2–7.2), *p* = 0.016. TAVI patients had more frequent early IE (3.2% vs. 0.3%, *p* = 0.006). In the PS analyses, IE risk did not differ: OR 0.65, 95% CI 0.32–1.32. Factors associated with TAVI IE included younger age (74y vs. 83y, *p* = 0.030), complicated diabetes mellitus (38.5% vs. 6.8%, *p* = 0.002), COPD (46.2% vs. 16.3%, *p* = 0.015), advanced heart failure (100% vs. 52.9%, *p* < 0.001), and peripheral arteriopathy (61.5% vs. 26.7%, *p* = 0.011). Conclusions: Early IE was higher with TAVI, but in the PS analyses, the risk attributable to each procedure was similar. Studies are needed to identify and optimize the risk factors of IE prior to TAVI.

## 1. Introduction

Infective endocarditis (IE) is one of the most feared complications after surgical aortic valve replacement (SAVR) [1,2]. It occurs in approximately 1.5% of patients after SAVR [3], and it is associated with poor outcomes [4]. 

In the last decades, transcatheter aortic valve replacement (TAVI) has been developed, and it is increasingly used for patients with high and prohibitive surgical risks [1]. Moreover, some trials have demonstrated its efficacy in low surgical risk patients [5]. The last clinical guidelines are increasingly accepting their use in this category [6]. Currently, there are controversial data on IE incidence after TAVI, but it is acknowledged that its occurrence is associated with higher mortality [7]. In clinical trials, including patients that could have been selected for both TAVI or SAVR, the incidence of IE after TAVI was around 1–1.5%, with no difference between procedures [3]. In some studies, based on administrative databases with short follow-up periods, IE incidence was also similar [4,8]. Nevertheless, in some real-world studies with complete follow-up data, some authors have found a higher incidence after TAVI [9,10]. 

Most of the factors associated with IE are more frequent in patients that undergo TAVI than in patients that undergo SAVR [4,7]. Consequently, patients selected for TAVI could present more frequently IE due to baseline differences with patients who are selected for SAVR. There is lacking evidence coming from prospective cohorts with large follow-up and baseline difference adjustments on the different IE risks for both procedures.

Our main aim was to evaluate the risk of IE after TAVI or SAVR and to determine if a potential difference in risk originates from different patient profiles selected for TAVI vs. SAVR. We also aimed to describe factors associated with IE among TAVI patients and to compare IE clinical presentation and outcomes between TAVI and SAVR patients. 

## 2. Material/Patients and Methods

We conducted a posthoc study based on a single-center prospective observational cohort. Our center is a 600-bed tertiary hospital with an attached population of 500,000 inhabitants. Our center is a referral hospital for complex cardiac surgeries and interventional cardiology procedures.

All patients aged 18 or older in whom an isolated aortic valve procedure was performed in our center from March 2015 to December 2020 were included in a prospective cohort. This cohort included both TAVI and SAVR procedures. Patients in whom the procedure was indicated for IE were excluded from this study.

We meticulously reviewed the electronic medical records of all patients included and identified IE episodes after the procedure. If one patient had more than one IE episode, we included only the first one. Baseline characteristics, procedure information, and clinician information on the IE episode were retrieved though electronic medical records. 

### 2.1. Procedure’s Methods and Follow-Up

TAVI procedure has been implemented in our institution since 2011 and is performed in a catheterization laboratory. Both TAVI and SAVR are carried out with maximum efforts for a sterile environment and with cefazolin prophylaxis by protocol. Follow-up was carried out for all patients in our medical outpatient clinic by hospital protocol. TAVI patients were followed-up at 1, 6, 12 months, and annually, SAVR patients at 3, 5, and 12 months and annually. 

### 2.2. Definitions

IE was defined according to modified Duke’s criteria [2]. IE occurring during the first 12 months of the procedure was considered early IE, and an episode occurring after 12 months was considered late IE. Surgery for the IE episode was indicated by the Endocarditis Team in accordance with current guidelines [2]. Sepsis and septic shock were defined as guidelines in use during the study period [11]. Local IE complications were defined as the presence of valve perforation, dehiscence, abscess, or pseudoaneurysm.

### 2.3. Primary and Secondary Outcomes

Our primary outcome was the incidence of IE after TAVI versus SAVR after adjusting for confounding variables using a propensity-score analysis (see below). The secondary outcome was identifying factors associated with IE among TAVI patients. We also compared IE episodes in TAVI patients versus SAVR patients. 

### 2.4. Statistical Analyses

Dichotomous variables are expressed as a percentage and absolute values. Continuous variables are expressed as the median and interquartile range (IQR). For inferential univariate analysis, the chi square test (or Fisher exact test if necessary) was used to compare dichotomous variables; and Mann–Whitney’s U was used to compare continuous variables. 

In order to estimate better the effect of either procedure on IE risk and to exclude confounding factors, a propensity score (PS) of being selected for TAVI (vs. SAVR) was created. To create the PS, we preselected variables previously identified in the literature as potentially associated both with IE after TAVI (vs. SAVR): age, Charlson index, diabetes mellitus, COPD, chronic renal failure, advanced heart failure, and EuroScore II [1,7,8,10,12,13]. These variables were included in a multivariate logistic regression model with the type of procedure as the dependent variable. Then, we calculated the inverse probability of treatment weight (IPTW) [14]. IPTW was stabilized by truncating it at the 95th percentile, and then an IPTW cohort (IPTWc) was formed. Balancing of the IPTWc was estimated by comparing PS distribution in the mentioned cohort, as well as comparing the absolute standardized mean difference (ASMD). An ASMD of less than 0.10 was considered optimal, whereas an SMD between 0.10 and 0.20 was considered acceptable [14]. The effect of TAVI vs. SAVR on IE risk was then estimated by means of logistic regression in the IPTWc. Odds ratio (OR) and 95% confidence interval (CI) are provided. Prespecified sensitivity analyses included a logistic regression model including the PS as a covariate in the total cohort, a multivariate logistic regression model including as covariates those variables with SMD greater than 0.10 in the IPTWc, and a double robust approach with generalized estimated equation in the IPTWc

Bilateral *p* values inferior to 0.05 were considered statistically significant. All statistical analyses were performed by means of the SPSS statistical software package (version 25, IBM, Armonk, New York, NY, USA).

#### 2.4.1. Ethical Statement

The study was approved by the hospital ethics committee (protocol 10/22). The study complied with the provisions in EU and Spanish legislation on data protection and the Declaration of Helsinki 2013. The informed consent for this study was waived because no extra procedures or visitations were made, and only previously generated data were recorded from medical electronic records. All patients signed the informed consent for the procedure.

#### 2.4.2. Patient and Public Involvement Statement

It was not appropriate and not possible to involve patients or the public in the design, conduct, reporting, and dissemination of our research.

## 3. Results

**Figure 1:**
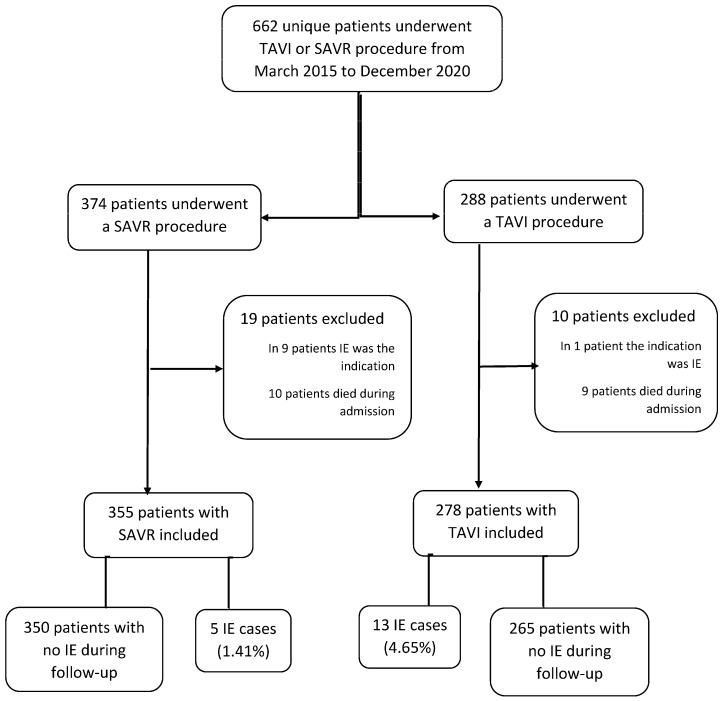
Patient’s flowchart. TAVI: Transcatheter aortic valve implantation. SAVR: Surgical aortic valve replacement. IE: infective endocarditis.

A total of 355 patients with SAVR and 278 with TAVI procedures were included. The patient’s flowchart is shown in Figure 1. Median follow-up was similar in both groups (38 months (26–51) vs. 41 (25–56), *p* = 0.550). During follow-up, a first IE episode occurred in 5 SAVR patients (1.41%, 95% CI 0.2–2.6) versus 13 TAVI patients (4.65%, 95% CI 2.2–7.2), *p* = 0.016. TAVI patients had more frequent early IE (3.2% (9 cases) vs. 0.3% (1 case), *p* = 0.006) but not late IE (1.4% (4 cases) vs. 1.1% (3 cases), *p* = 0.731). The baseline characteristics of TAVI and SAVR patients are compared in Table 1.

### 3.1. Propensity Score

**Figure 2:**
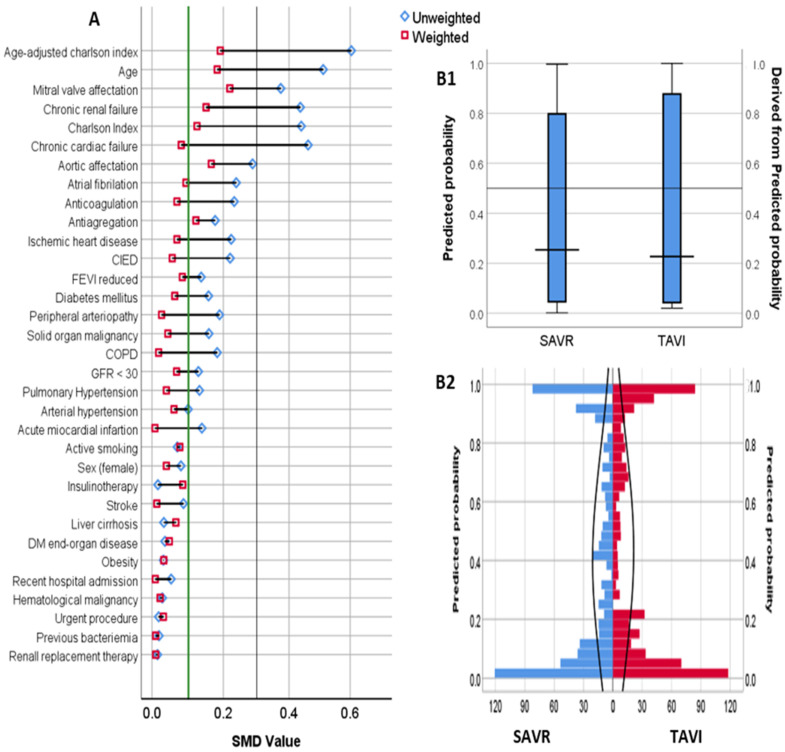
Covariates and propensity score distribution among IPTW cohort. (**A**): Standardized mean difference (SMD) among baseline covariates. Green line represents SMD of 10%. Blue dot represents the SMD value in the unweighted cohort. Red dot represents the value in the Inverse Probability of Treatment Weighted cohort. B: Propensity score of TAVI numeric (**B1**) and absolute (**B2**) distribution among patients with TAVI and SAVR in the IPTW cohort.

A PS for TAVI vs. SAVR was created, including mentioned preselected variables. Table 2 summarizes the multivariate logistic regression model for PS calculation. Statistic C for the PS was 0.940 (95% CI 0.92–0.96). After weighting, both groups were generally well-balanced for baseline characteristics and comorbidity in the IPTWc (Table 1 and Figure 2). 

### 3.2. Primary Outcome: Effect of TAVI vs. SAVR

**Figure 3:**
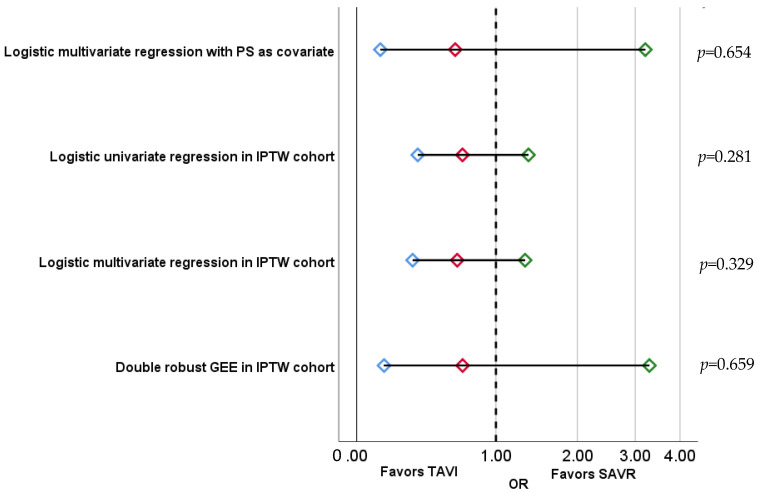
Association between aortic valve procedure and infective endocarditis after adjusting for cofounders with propensity score in different sensitivity analyses. Red dot indicates the Odds Ratio (OR), and the interval between blue and green dots indicates 95% confidence interval for the OR. TAVI: Transcatheter aortic valve implantation. SAVR: Surgical aortic valve replacement.

In the IPTWc, IE risk after TAVI versus SAVR did not differ: OR 0.65, 95% CI 0.23–1.32, *p* = 0.329. There was no association between the type of procedure and IE risk in other prespecified sensitivity analyses (Figure 3). 

### 3.3. Factors Associated with IE among TAVI Patients

Table 3 summarizes factors associated with IE in TAVI patients. IE patients were younger (73 years (70–84) vs. 83 (77–87), *p* = 0.030) and had more frequent comorbidities, including peripheral arteriopathy (61.5% vs. 26.7%), COPD (46.2% vs. 16.3%, *p* = 0.011) and advanced heart failure (100% vs. 52.9%, *p* < 0.001). 

Due to low number of events (IE cases), no statistical analyses were performed to search for factors associated with IE among SAVR cases.

### 3.4. IE Characteristics in Patients with TAVI vs. SAVR

Table 4 summarizes IE characteristics, microbiology, and outcomes in TAVI and SAVR patients. TAVI patients with IE had more comorbidities (Charlson index 7 (6–9) vs. 5 (4–5), *p* = 0.026) and a shorter period from procedure to IE (30 weeks (6–70) vs. 50 (18–148), *p* = 0.005). Clinically, TAVI patients tended to have a less frequent fever without reaching statistical significance (58.3% vs. 100%, *p* = 0.086). There were no differences in other clinical characteristics and diagnostic work-up. The most frequent microorganism causing post-TAVI IE was *Enterococcus faecalis* (38.5%), followed by *Staphylococcus aureus* (30.8%) and coagulase-negative staphylococci (23.1%). Meanwhile, the microorganisms causing post-SAVR IE were *Staphylococcus aureus* (20%), coagulase-negative staphylococci (20%), *Streptococcus gallolyticus* (20%), *Streptococcus viridans* (20%), and *Enterococcus faecalis* (20%). Both groups presented a similar prevalence of surgical indication (41.7% (5) vs. 40.0% (2), *p* = 1.000). None of the 5 TAVI patients with surgical indication underwent surgery vs. 2 of the 2 patients with a surgical indication among SAVR were intervened (*p* = 0.086). In-hospital and 1-year mortality were similar in both groups (41.7% vs. 60.0%, *p* = 1.000 and 58.3% vs. 60.0%, *p* = 1.000, respectively).

Of note, among patients with IE post-TAVI, those with surgical indication for the IE had higher in-hospital mortality than those with no surgical indication (80.0% (4/5) vs. 0% (0/7), *p* = 0.01). The reasons for rejecting surgery were comorbidities and technical complexity of the procedure (n = 3) and poor clinical situation (n = 2). The patient with surgical indication who did not die during admission presented an IE recurrence caused by the same microorganism (*Enterococcus faecalis*). Surgery was performed during the second episode. The patient was discharged with no further complications. Another patient with *Enterococcus faecalis* IE with no surgical indication presented a late IE recurrence caused by *Streptococcus oralis*. There was no recurrence among SAVR patients. 

## 4. Discussion

In our study, our main finding is that TAVI patients had more frequent IE, especially early IE, but it was related to the greater patient’s baseline IE risk. The risk of IE associated with both procedures was comparable after adjusting for possible confounding variables in the PS analyses.

IE rates in our SAVR patients were similar as described in recent studies [15,16,17], but TAVI IE, especially early IE, was higher in comparison to reports from clinical trials [3]. Our TAVI patients had more comorbidities and higher surgical risk in comparison with most trials [5,18,19,20]. Both peripheral arteriopathy and chronic renal failure were found in greater prevalence in our patients. Our cohort also had slightly more comorbidities than patients included in trials considering only high-risk patients [21]. We had younger patients, which has been associated with post-TAVI IE [4,7]. The association between younger age and post-TAVI IE has been related to the higher burden of comorbidity that may have younger patients elected for TAVI [21]. Still, our post-TAVI IE incidence was similar to a recent study publishing a 5-year follow-up on these patients (3.7%) [20]. Other studies with real-world data and complete follow-up have reported a similar incidence as ours [9]. IE characteristics, microbiology, and outcomes among TAVI patients were quite similar to what was previously described, including early IE predominance [12,22,23,24,25], vascular foci with nosocomial or healthcare-acquired onset [7,9], predominance of *Enterococcus faecalis* [10,25], and a lower frequency of fever [13]. This different clinical profile of IE after TAVI vs. SAVR has been related to a greater age, comorbidity, and femoral access as the original source of infection [10,22,25]. In-hospital and 1-year mortality rates were similar to what was previously described [10,22,23,26]. We hypothesize that our higher IE rate was consequence of more complex patients and the fact that we performed a close and long follow-up in all patients, allowing us to diagnose more cases than other studies with shorter follow-up periods. 

On the other hand, among patients who were selected for TAVI, we were able to find several factors associated with IE. Including patient’s complexity (measured by means of the Charlson index), complicated diabetes mellitus, COPD, cardiac failure, peripheral arteriopathy, chronic renal failure, and recent hospital admission. These factors have also been described by other authors [1,7,8,10,12,13] Yet, we did not recognize some other factors that have been previously described, such as Surgical risk (EuroScore II), probably due to our relatively low sample size [4].

Some of the mentioned factors could have influenced the decision to perform TAVI instead of SAVR, which would advocate a higher IE risk in patients selected for TAVI. Subsequently, after weighting for the preselected variables in the propensity-score analyses, the risk of IE attributable to the procedure itself was similar for TAVI and SAVR (OR 0.65, 95% CI 0.23–1.32), with similar results in sensitivity analyses. These results are similar to what other authors found in population-based studies [4,8]. However, these studies have biases, such as incomplete and/or inaccurate data from administrative databases. Hence, our results provide confirmation of what is previously described and suggest that the risk of IE attributable to SAVR and TAVI procedures themselves are similar and comparable. 

Separately, we noticed that most of the patients with post-TAVI IE and surgical indications were rejected for surgery. This assessment was mainly based on previous comorbidities and technical complexity, and it could have influenced our outcomes. Other authors have also noted low surgical rates in patients with post-TAVI IE [1,9,27]. Of note, one patient of our cohort with surgical indication who survived the episode was later operated on in a second IE episode with no complications. Other authors have shown the importance of a multidisciplinary approach, within an Endocarditis team, in evaluating the surgical risk of IE patients and optimizing surgical decisions [28,29,30]. We speculate that patients with IE after TAVI should not be discarded for surgery based only on their comorbidity and complexity. Rather, a careful assessment by the Endocarditis Team should be carried out, often with decision revaluation during the hospitalization.

## 5. Limitations

This is a single-center observational study with inherent limitations. Our main limitation is our relatively low sample size, which may have limited the statistical power of the study. However, our results are in accordance with what was found by other authors, which procures credibility to our findings. Secondly, we cannot discard that some patients have been diagnosed with IE in their original hospitals. However, this risk should be minimal due to the close follow-up visitations performed at our center. Finally, as our work is an observational study, we are cautious in extracting causal relationships. Nevertheless, our data sum to the body of evidence that suggests that the risk of IE after TAVI is comparable to the risk after SAVR. 

## 6. Conclusions

IE rate in TAVI is higher than IE after SAVR, particularly early IE. TAVI patients present a higher baseline risk due to higher comorbidity burden, especially diabetes mellitus, COPD, advanced heart failure, and peripheral arteriopathy. In the propensity-score analyses, the IE risk attributable to each procedure was comparable. More studies are needed to identify and optimize risk factors of IE prior to either procedure and to define surgery indications and contraindications in these patients.

## Figures and Tables

**Table 1:** Baseline characteristics and IR occurrence after TAVI vs. SAVR. TAVI: Transcatheter aortic valve implantation. SAVR: Surgical aortic valve implantation. CI: Charlson Index. COPD: Chronic obstructive pulmonary disease. CIED: Cardiac Implantable Electronic Device. GFR: Glomerular filtration rate. IE: Infective endocarditis. AMSD: Absolute mean standardized difference.

Variable	Unadjusted Model				PS-Adjusted (IPTW)	Miss
	TAVI (*n* = 279)	SAV (*n* = 355)	*p*	AMSD	AMSD	
Age (years)	83 (77–86)	69 (61–75)	<0.001	0.510	0.182	0
Sex (female)	48.8% (123)	36.3% (129)	0.050	0.079	0.039	0
Charlson Index	2 (1–4)	1 (0–2)	<0.001	0.439	0.124	4
Age-adjusted CI	6 (5–8)	3 (2–4)	<0.001	0.604	0.191	
Active smoking	4.7% (13)	11.5% (41)	0.001	0.069	0.076	6
Arterial hypertension	79.5% (221)	69.6% (247)	0.006	0.099	0.060	1
Diabetes mellitus	42.4% (118)	26.8% (95)	<0.001	0.157	0.062	1
*End-organ disease*	8.3% (23)	4.8% (17)	0.099	0.035	0.047	2
*Insulin therapy*	7.6% (21)	5.9% (21)	0.425	0.016	0.084	
Mitral valve function (normal)	37.0% (102)	74.4% (264)	<0.001	0.374	0.219	3
COPD	17.8% (49)	9.6% (34)	0.003	0.182	0.018	3
Atrial fibrillation	38.5% (107)	14.7% (52)	<0.001	0.238	0.093	2
Advanced heart failure	55.1% (152)	18.0% (64)	<0.001	0.461	0.080	3
*Reduced FEVI*	29.2% (81)	15.5% (55)	<0.001	0.136	0.083	4
Pulmonary hypertension	38.1% (106)	25.1% (89)	0.001	0.131	0.039	1
Ischemic heart disease	34.4% (95)	12.1% (43)	<0.001	0.223	0.068	3
CIED	25.9% (72)	3.9% (14)	<0.001	0.220	0.055	1
Peripheral arteriopathy	28.5% (79)	9.6% (34)	<0.001	0.189	0.026	3
Chronic renal failure	54.3% (151)	10.7% (38)	<0.001	0.436	0.150	1
GFR < 30 mg/mL/min	15.1% (42)	2.3% (8)	<0.001	0.128	0.067	2
Renal replacement therapy	1.8% (5)	0.3% (1)	0.092	0.015	0.010	2
Liver cirrhosis	5.8% (16)	2.5% (9)	0.042	0.032	0.065	1
Stroke	15.1% (42)	6.5% (23)	<0.001	0.086	0.013	1
Obesity	21.9% (61)	25.1% (89)	0.397	0.031	0.032	1
Active solid malignancy	16.9% (47)	1.1% (4)	<0.001	0.158	0.043	1
Hematological malignancy	3.6% (10)	0.9% (3)	0.007	0.028	0.022	1
Anticoagulation	39.9% (110)	26.6% (59)	<0.001	0.232	0.068	3
Antiaggregation	44.6% (124)	27.0% (96)	<0.001	0.176	0.121	1
Recent hospital admission	13.1% (36)	7.9% (28)	0.034	0.052	0.009	4
Previous bacteremia	3.0% (8)	1.1% (4)	0.138	0.019	0.010	13
Urgent procedure	9.1% (25)	7.0% (25)	0.453	0.018	0.031	4
Euroscore II (%)	5.5 (3.2–8.8)	1.4 (0.8–2.3)	<0.001	0.598	0.178	6
Infective endocarditis	4.7% (13)	1.4% (5)	0.017		-	0
Early IE	3.2% (9)	0.3% (1)	0.006		-	0
Late IE	(4)	(3)	0.705		-	0
Follow-up (months)	41 (25–56)	38 (25–55)	0.555		-	0

Italics to differentiate the types of DM (End organ and DM insulin therapy).

**Table 2:** Multivariate logistic regression model for creating the propensity score of TAVI vs. SAVR procedure. OR: odds ratio. 95% CI: 95% confident interval. TAVI: transcatheter aortic valve implantation. SAVR: Surgical aortic valve replacement.

Variable	OR	95% CI	*p*
Age (per 5 years)	2.37	1.91–2.94	<0.001
Charlson Index (per point)	1.25	1.05–1.48	0.013
Diabetes mellitus	3.66	1.35–10.0	0.011
Chronic obstructive pulmonary disease	1.18	0.60–2.32	0.631
Advanced heart failure	10.10	5.26–19.38	<0.001
Chronic renal failure	3.17	1.76–5.71	<0.001
Euroscore II (per 1% of risk increment)	1.27	1.09–1.48	0.002

**Table 3:** Factors associated with IE among patients with TAVI. IE: Infective endocarditis. TAVI: transcatheter aortic valve implantation. COPD: Chronic obstructive pulmonary disease. CIED: Cardiac implantable electronic device. GFR: Glomerular filtration rate.

Variable	IE (*n* = 13)	No IE (*n* = 265)	*p*	Missing
Age (years)	74 (70–84)	83 (77–87)	0.030	0
Sex (female)	30.8% (4)	44.9% (119)	0.398	0
Charlson Index	4 (2–6)	2 (1–4)	0.089	3
Age-adjusted Charlson Index	7 (5–8)	6 (5–7)	0.178	3
Active smoking	0	5.0% (13)	0.365	5
Arterial hypertension	92.3% (12)	78.9% (209)	0.316	1
Diabetes mellitus	53.8% (7)	41.9% (111)	0.567	1
*No cardiac end-organ disease*	38.5% (5)	6.8% (18)	0.002	2
*Insulin therapy*	30.8% (4)	6.4% (17)	0.011	1
Valve replacement indication	-	-	0.212	3
*Aortic stenosis*	84.6% (11)	65.4% (172)		
*Aortic insufficiency*	0	1.5% (4)		
*Double aortic lesion*	15.4% (2)	33.1% (87)		
COPD	46.2% (6)	16.3% (43)	0.015	3
Atrial fibrillation	38.5% (5)	38.5% (5)	1.000	1
Advanced heart failure	100% (13)	52.9% (139)	<0.001	3
*Reduced LVEF*	53.8% (7)	27.8% (74)	0.048	1
Pulmonary hypertension	61.5% (8)	37.0% (98)	0.037	1
Ischemic heart disease	23.1% (3)	35.0% (92)	0.552	3
*Acute ischemic heart disease*	23.1% (3)	20.8% (55)	1.000	1
CIED	38.5% (5)	25.3% (67)	0.322	1
Peripheral arteriopathy	61.5% (8)	26.7% (71)	0.011	2
Chronic renal failure	84.6% (11)	52.8% (140)	0.042	1
*GFR < 30 mg/mL/min*	23.1% (3)	14.7% (39)	0.424	1
*Renal replacement therapy*	0	1.9% (5)	1.000	1
Liver cirrhosis	7.7% (1)	5.7% (15)	1.000	1
Stroke	30.8% (4)	14.3% (38)	0.115	1
Obesity	30.8% (4)	21.5% (57)	0.491	1
Active malignancy	15.4% (2)	17.0% (45)	1.000	1
Anticoagulation	46.2% (6)	39.5% (107)	0.774	3
Antiaggregation	46.2% (6)	44.5% (119)	1.000	1
Recent hospital admission	53.8% (7)	11.1% (29)	<0.001	4
Previous bacteremia	20.0% (2)	2.3% (6)	0.041	7
Day hospital follow-up	20.0% (2)	16.1% (38)	1.000	33
Urgent procedure	7.7% (1)	9.2% (24)	1.000	4
Euroscore II (%)	6.3 (2.0–13.3)	5.5 (3.2–8.5)	0.786	5
Transfemoral venous access	92.3% (12)	95.8% (228)	0.450	28
Postprocedural valvular leak	53.8% (7)	40.8% (107)	0.485	4
Max. aortic gradient	18 (13–25)	19 (15–25)	0.647	
Medium aortic gradient	9 (7–13)	10 (8–13)	0.566	
Postprocedural complication	30.8% (4)	42.5% (111)	0.567	5
Length of hospital admission (days)	5 (2–11)	5 (4–7)	0.968	

**Table 4:** Baseline characteristics, clinical presentation, diagnostic work-up, and outcomes of IE in patients with TAVI versus SAVR.

Variable		TAVI (n = 13)	SAVR (n = 5)	*p*
**Baseline characteristics and comorbidites**				
Age (years)		75 (70–85)	77 (63–79)	0.377
Sex (female)		30.8% (4)	0	0.278
Charlson index		4 (2–6)	2 (0–3)	<0.001
Age-adjusted Charlson index		7 (6–9)	5 (4–5)	0.026
Arterial hypertension		92.3% (12)	60.0% (3)	0.172
Diabetes mellitus		53.8% (7)	40.0% (2)	1.000
Chronic pulmonary obstructive disease		46.2% (6)	20.0% (1)	0.596
Advanced heart failure		100% (13)	20.0% (1)	0.002
Ischemic heart disease		23.1% (3)	60.0% (3)	0.268
Peripheral artery disease		46.2% (6)	0	0.063
Chronic renal failure		84.6% (11)	20.0% (1)	0.022
**Clinical presentation**				
Time from valve procedure (weeks)		30 (6–70)	50 (18–148)	0.005
Early infective endocarditis		76.9% (10)	20.0% (1)	0.074
Acquisition	Community	23.1% (3)	60.0% (3)	0.281
	Nosocomial	38.5% (5)	40.0% (2)	
	HCA	38.5% (5)	0	
Primary focus	Unknown	30.8% (4)	0	0.024
	Vascular	46.2% (6)	0	
	Other	24.0% (3)	100% (5)	
Symptoms duration *	Less 2 weeks	60.0% (6)	100% (5)	0.286
	2–4 weeks	20.0% (2)	0	
	More 1 month	20.0% (2)	0	
Fever		58.3% (7)	100% (5)	0.086
Acute heart failure		41.7% (5)	40.0% (2)	1.000
Acute renal injury		58.3% (7)	60.0% (3)	1.000
Septic shock		33.3% (4)	60.0% (3)	0.593
Embolisms		30.8% (4)	20.0% (1)	1.000
Persistent bacteremia		50.0% (5)	0	0.078
Local valve complication		23.1% (3)	20.0% (1)	1.000
**Diagnostic work-up**				
Positive transthoracic echocardiogram		40.0% (4/10)	25.0% (1/4)	0.510
Positive transesophageal echocardiogram		58.3% (7/12)	60.0% (3/5)	1.000
Positive PET-CT		100% (6/6)	100% (3/3)	1.000
**Microbiology**				
*Staphylococcus aureus*		30.8% (4)	20.0% (1)	1.000
Coagulase-negative staphylococci		23.1% (3)	20.0% (1)	1.000
*Enterococcus faecalis*		38.5% (5)	20.0% (1)	0.205
*Streptococcus* spp.		7.7% (1)	40.0% (2)	0.172
**Management and outcomes**				
Cardiac surgery indication		41.7% (5)	40.0% (2)	1.000
Cardiac surgery performed		0	40.0% (2)	0.095
Cardiac surgery indicated and not performed		41.7% (5)	0	0.086
In-hospital mortality		41.7% (5)	60.0% (3)	1.000
1-year mortality		58.3% (7)	60.0% (3)	1.000
Recurrence		28.6% (2/7)	0/2	0.444

* Three patients among TAVI had unknown symptoms duration.

## Data Availability

The data underlying this article are available in the article.

## Abbreviations

IE	Infective endocarditis
SAVR	Surgical aortic valve replacement
TAVI	Transcatheter aortic valve implantation
COPD	Chronic obstructive pulmonary disease
IPTW	Inverse probability of treatment weight
IPTWc	Inverse probability of treatment weight cohort
ASMD	Absolute standardized mean difference
PS	Propensity score
